# Phosphosilicate Fiber-Based Low Quantum Defect Raman Fiber Laser with Ultrahigh Spectral Purity

**DOI:** 10.3390/nano12091490

**Published:** 2022-04-27

**Authors:** Yang Zhang, Jiangming Xu, Sicheng Li, Junrui Liang, Jun Ye, Xiaoya Ma, Tianfu Yao, Pu Zhou

**Affiliations:** College of Advanced Interdisciplinary Studies, National University of Defense Technology, Changsha 410073, China; zhangyang18a@nudt.edu.cn (Y.Z.); lisicheng12@nudt.edu.cn (S.L.); liangjunrui17@nudt.edu.cn (J.L.); yejun12@nudt.edu.cn (J.Y.); maxiaoya19@nudt.edu.cn (X.M.); yaotianfu@nudt.edu.cn (T.Y.)

**Keywords:** quantum defect, phosphosilicate fiber, Raman fiber laser

## Abstract

The phosphosilicate fiber-based Raman fiber laser (RFL) has great potential in achieving low-quantum defect (QD) high-power laser output. However, the laser’s performance could be seriously degraded by the Raman-assisted four-wave mixing (FWM) effect and spontaneous Raman generation at 14.7 THz. To find possible ways to suppress the Raman-assisted FWM effect and spontaneous Raman generation, here, we propose a revised power-balanced model to simulate the nonlinear process in the low-QD RFL. The power evolution characteristics in this low-QD RFL with different pump directions are calculated. The simulation results show that, compared to the forward-pumped low-QD RFL, the threshold powers of spontaneous Raman generation in the backward-pumped RFL are increased by 40% and the Raman-assisted FWM effect is well suppressed. Based on the simulation work, we change the pump direction of a forward-pumped low-QD RFL into backward pumping. As a result, the maximum signal power is increased by 20% and the corresponding spectral purity is increased to 99.8%. This work offers a way for nonlinear effects controlling in low-QD RFL, which is essential in its further performance scaling.

## 1. Introduction

Quantum defect (QD), as a troublesome issue in fiber lasers, not only limiting the conversion efficiency, but more importantly, being the main source of thermal load in fiber lasers. Thermal load, however, could result in many deleterious effects such as transverse mode instability, thermal noise and even fiber fusing [1,2,3,4,5], thus threatening the safety and stability of fiber lasers. Therefore, it is of great importance to reduce QD in fiber lasers. In past decades, lots of low-QD fiber lasers have been reported, most of which are based on rare-earth-doped fibers [6,7,8,9,10,11,12,13,14]. For example, in 2009, researchers from IPG reported a 10 kW fiber laser with a QD of 4.86% through tandem pumping [15], while the QDs of common ytterbium-doped fiber lasers are generally around 10% (pumped at 976 nm and emitted at 1070 or 1080 nm). In 2018, Yu et al. demonstrated hundred-milliwatt-level laser output with less than 1% QD via specially designed ytterbium-doped multicomponent fluorosilicate fiber [16]. Restricted by the absorption and emission spectra of rare-earth ions, it is inherently difficult to achieve low-QD laser output with high output power in rare-earth-doped fiber lasers. Besides, passive fiber-based Raman fiber laser (RFL) is also a great candidate for low-QD laser generation [17,18,19]. In common silica fiber-based RFL pumped at 1070 nm and operating at 1123 nm (peak Raman gain), the QD is about 4.7%, similar to that of tandem pumped ytterbium-doped fiber laser [18]. It can be further reduced by exploring the RFL to work under smaller frequency shifts, at the sacrifice of Raman gain and output power [20,21].

Recently, we found that the phosphosilicate fiber-based RFL has great potential in achieving ultra-low-QD high-power laser output [22]. Different from common silica fiber, the phosphosilicate fiber has a strong boson peak in the low-frequency shift area. By adopting the boson peak to provide Raman gain, we have reported hundred-watt level output with less than 1% QD [23]. However, further power scaling and spectral purity is limited by the Raman-assisted four-wave mixing (FWM) effect and spontaneous Raman generation at 14.7 THz [22,23,24]. In conventional silica fiber-based RFL, the frequency shift between the pump and Raman signal is 13.2 THz, the FWM effect is well suppressed due to the dispersion induced phase mismatch and walk-off effect [25,26]. However, in the low-QD fiber laser, the frequency shift between the pump and Raman signal is reduced to 3.65 THz, and the differences in the propagation constant and group velocity between the pump light and Raman signal are much smaller. Consequently, FWM processes could take place under the assistance of Raman amplification [27,28], thus restricting the further power scaling of low-QD RFL.

In this paper, we propose a revised power-balanced model to simulate the Raman-assisted FWM and spontaneous Raman generation process in phosphosilicate fiber-based low-QD RFL. The simulation results show that compared to the forward-pumped low-QD RFL, the spontaneous Raman generation and the Raman-assisted FWM effect is well suppressed in the backward pumped RFL. Based on the simulation work, we change the pump direction of a forward pumped low-QD RFL into backward pumping. As a result, the maximum signal power is increased by 20% and the corresponding spectral purity is increased from 94.2% to 99.8%.

## 2. Theoretical Analysis

To verify the backward pumped low-QD RFL’s advantage in suppressing spontaneous Raman generation at 14.7 THz, a revised power-balanced model is proposed to calculate the power evolution characteristics of our low-QD RFL. Different from the general power-balanced model of conventional silica fiber-based RFL [29,30], in the low-QD RFL, the pump power can be converted into the 1080 nm signal light and spontaneous Raman light at 14.7 THz. Meanwhile, the 1080 nm signal light can also be converted to the spontaneous Raman light at 14.7 THz. Consequently, the power evolution process in the low-QD RFL can be described as follows:

(1) $$\pm \frac{dP_{0}^{\pm }}{\mathrm{dz}}=-\alpha_{0}P_{0}^{\pm }-\frac{g_{R01}}{A_{eff}}\frac{\upsilon_{0}}{\upsilon_{1}}P_{0}^{\pm }(P_{1}^{+}+P_{1}^{-}+\Gamma_{1})-\frac{g_{R02}}{A_{eff}}\frac{\upsilon_{0}}{\upsilon_{2}}P_{0}^{\pm }(P_{2}^{+}+P_{2}^{-}+\Gamma_{2})+\varepsilon_{0}P_{0}^{\mp },$$

(2) $$\pm \frac{dP_{1}^{\pm }}{\mathrm{dz}}=-\alpha_{1}P_{1}^{\pm }+\frac{g_{R01}}{A_{eff}}(P_{1}^{\pm }+\frac{\Gamma_{1}}{2})(P_{0}^{+}+P_{0}^{-})-\frac{g_{R12}}{A_{eff}}\frac{\upsilon_{1}}{\upsilon_{2}}P_{1}^{\pm }(P_{2}^{+}+P_{2}^{-}+\Gamma_{2})+\varepsilon_{1}P_{1}^{\mp },$$

(3) $$\pm \frac{dP_{2}^{\pm }}{\mathrm{dz}}=-\alpha_{2}P_{2}^{\pm }+\frac{g_{R12}}{A_{eff}}(P_{2}^{\pm }+\frac{\Gamma_{2}}{2})(P_{1}^{+}+P_{1}^{-})+\frac{g_{R02}}{A_{eff}}(P_{2}^{\pm }+0.5\Gamma_{2})(P_{0}^{+}+P_{0}^{-})+\varepsilon_{2}P_{2}^{\mp },$$

(4) $$\Gamma_{j}=4h\upsilon_{j}\Delta \upsilon_{j}\left\{1+\frac{1}{\exp\left[h(\upsilon_{j-1}-\upsilon_{j})/k_{B}T\right]-1}\right\},\;j=1,\;2,$$

here, lower indexes 0, 1, 2 refer the corresponding terms to pump, 1080 nm signal light and spontaneous Raman light at 14.7 THz, while upper indexes + and − refer to forward and backward propagating waves, respectively. *P* corresponds to the power, *α* defines the attenuation of the corresponding wave, *ν* is the frequency, Δ*ν* is the Raman amplification bandwidth, *ε* is the backscattering coefficient, derived as *α* multiplied by backscattering factor *Q*, *ε = α* * *Q*, *ε* = *α* * *Q*, with *Q* equal approximately to 0.0017 for the phosphosilicate fiber [31]. T (= 298 K) is the absolute temperature, *A_eff_* is the effective mode area, *k_B_* is the Boltzmann’s constant, *h* is the Planck’s constant. *g_R_*_01_, *g_R_*_02_, *g_R_*_12_, correspond to the Raman gain coefficient from pump to signal, pump to spontaneous light at 14.7 THz, and signal to spontaneous light at 14.7 THz, respectively. The boundary conditions are as follows:

(5) $$P_{0}=P_{in},$$

(6) $$P_{j}^{+}(0)=P_{j}^{-}(0)R_{Lj},\;j=1,\;2,$$

(7) $$P_{j}^{-}(L)=P_{j}^{+}(L)R_{Rj},\;j=1,\;2.\;$$

Based on the proposed power-balanced model above, we calculate the power evolution characteristics of the forward-pumped low-QD RFL and backward-pumped low-QD RFL. Fiber parameters in our numerical calculation are summarized in Table 1. In the forward-pumped low-QD RFL, *R_L_*_1_ is 0.99 and *R_R_*_1_ is 0.10. This is reversed in the backward pumped low QD RFL, where *R_L_*_1_ is 0.10 and *R_R_*_1_ is 0.99. Moreover, considering the possible tiny parasitic reflection on fiber ends in the experiment, *R_L_*_2_ and *R_R_*_2_ are both set at 2 × 10^−5^.

**Figure 1:**
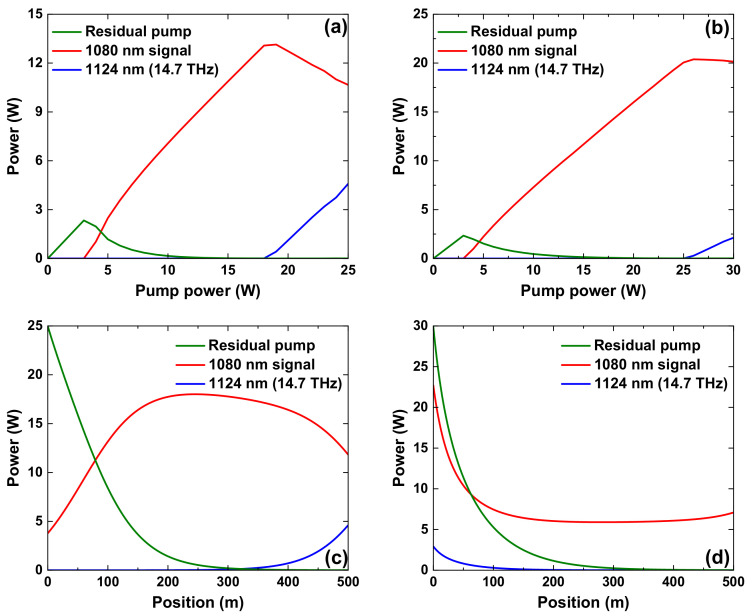
The power evolution characteristics of (**a**) forward-pumped low-quantum defect Raman fiber laser and (**b**) backward-pumped low-quantum defect Raman fiber laser; The power distribution characteristics of (**c**) forward-pumped low-quantum defect Raman fiber laser and (**d**) backward-pumped low-quantum defect Raman fiber laser.

The calculated power characteristics of the forward-pumped low-QD RFL and backward pumped low-QD RFL are displayed in the following figure. Figure 1a,b show the power evolution characteristics of the forward-pumped low-QD RFL and backward-pumped low-QD RFL, respectively. In the backward-pumped low-QD RFL, the threshold power of spontaneous Raman generation at 14.7 THz is 25 W, 39% higher than the 18 W in the forward-pumped low-QD RFL. Moreover, the maximum output signal power is 20.36 W in the backward-pumped low-QD RFL, 55% higher than the 13.14 W in the forward-pumped low-QD RFL. Figure 1c,d show the power distribution characteristics of the forward-pumped low-QD RFL and backward-pumped low-QD RFL, respectively. It can be seen that in the forward-pumped RFL, the 1080 nm signal light is amplified in the former section of the fiber. The high-power 1080 nm signal light has to pass through a long section of passive fiber before being outputted. During this process, part of the 1080 nm signal light could be converted into Raman light at 1124 nm due to the stimulated Raman scattering effect. In the backward-pumped RFL, the 1080 nm signal light is mainly amplified near the output port. The transmission distance of the 1080 nm signal light before being outputted is significantly shorter than that in the forward-pumped RFL. Consequently, less 1080 nm signal light is converted into the Raman light at 1124 nm, which accounts for the higher threshold power of spontaneous Raman generation at 14.7 THz and higher output power at 1080 nm in backward-pumped low-QD RFL.

**Figure 2:**
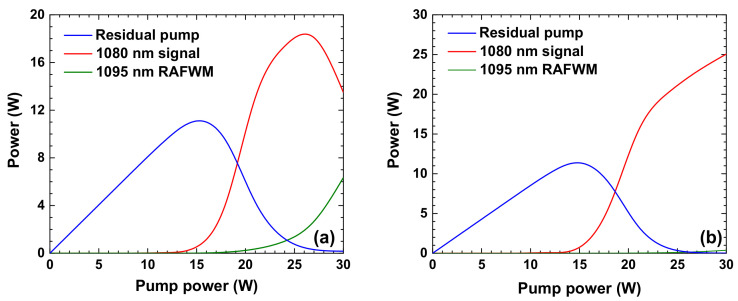
The power evolution characteristics of the Raman-assisted FWM process in the (**a**) forward-pumped low-QD Raman amplifier and (**b**) backward-pumped low-QD Raman amplifier. RAFWM, Raman-assisted four-wave mixing.

Moreover, the Raman-assisted FWM process in the low-QD RFL is also investigated. During the Raman-assisted FWM process, the FWM effect-generated peak at 1095 nm will experience subsequent Raman amplification from the 1066 pump source and 1080 nm signal light. Given that the FWM process is a phase-sensitive process, we consider a Raman amplifier where the thermal noise and backward scattering is not considered for the simplification. The power-balanced model for the Raman-assisted FWM process in the low-QD Raman amplifier is as follows [32,33]:

(8) $$\frac{\partial P_{0}}{\partial z}=-2\frac{n_{2}\omega_{0}}{cA_{eff}}P_{1}\sqrt{P_{3}P_{0}}\sin\theta -\frac{g_{R01}}{A_{eff}}\frac{\upsilon_{0}}{\upsilon_{1}}P_{0}P_{1}-\frac{g_{R03}}{A_{eff}}\frac{\upsilon_{0}}{\upsilon_{3}}P_{0}P_{3}-\alpha_{0}P_{0},$$

(9) $$\frac{\partial P_{1}}{\partial z}=4\frac{n_{2}\omega_{1}}{cA_{eff}}P_{1}\sqrt{P_{3}P_{0}}\sin\theta -\frac{g_{R13}}{A_{eff}}\frac{\upsilon_{1}}{\upsilon_{3}}P_{1}P_{3}+\frac{g_{R01}}{A_{eff}}P_{0}P_{1}-\alpha_{1}P_{1},$$

(10) $$\frac{\partial P_{3}}{\partial z}=-2\frac{n_{2}\omega_{3}}{cA_{eff}}P_{1}\sqrt{P_{3}P_{0}}\sin\theta +\frac{g_{R13}}{A_{eff}}P_{1}P_{3}+\frac{g_{R03}}{A_{eff}}P_{0}P_{3}-\alpha_{3}P_{3},$$

here, the lower indexes 0, 1, 3 refer the corresponding terms to pump, 1080 nm signal light and FWM effect generated peak at 1095 nm. n_2_ is the nonlinear-index coefficient. *θ* is the relative phase difference between these three waves, and it is governed by:

(11) $$\frac{\partial \theta }{\partial z}=\Delta k+2\frac{n_{2}}{cA_{eff}}\left(2\omega_{1}\sqrt{P_{3}P_{0}}-\omega_{3}\frac{P_{1}}{2}\sqrt{\frac{P_{0}}{P_{3}}}-\omega_{0}\frac{P_{1}}{2}\sqrt{\frac{P_{3}}{P_{0}}}\right)\cos\theta +\frac{n_{2}}{cA_{eff}}\left(\begin{array}{l} \omega_{1}\left[P_{1}+\left(2-\rho \right)\left(P_{1}+2P_{3}+2P_{0}\right)\right]\\ -\omega_{3}\left[P_{3}+\left(2-\rho \right)\left(P_{1}+P_{0}\right)\right]\\ -\omega_{0}\left[P_{0}+\left(2-\rho \right)\left(P_{1}+P_{3}\right)\right] \end{array}\right),$$

(12) $$\Delta k=2\beta_{1}-\beta_{0}-\beta_{3},$$

where *β* is the is the propagation constant of the light wave, Δ*k* is the propagation constant difference and is set at 0.5 m^−1^ in the simulation. *ρ* is fractional Raman contribution, typically 0.18 [32]. Based on the proposed power-balanced model above, we calculate the power evolution characteristics of a forward-pumped low-QD Raman amplifier and backward-pumped low-QD Raman amplifier. To be noted, in the backward-pumped low-QD Raman amplifier, the pump light and Raman signal propagate in different directions and the FWM process is well suppressed, thus the FWM process is not considered in the backward-pumped low-QD Raman amplifier. The power evolution characteristics of these two laser systems are shown in Figure 2 as follows. In the forward-pumped low-QD Raman amplifier, the threshold power of Raman-assisted FWM-related light at 1095 nm is around 18 W, while in the backward-pumped low-QD Raman amplifier, the threshold power of the light component at 1095 nm is increased to 27 W. The results confirm the advantage of the backward pump scheme in suppressing the Raman-assisted FWM process in low-QD RFL.

## 3. Experimental Setup

**Figure 3:**
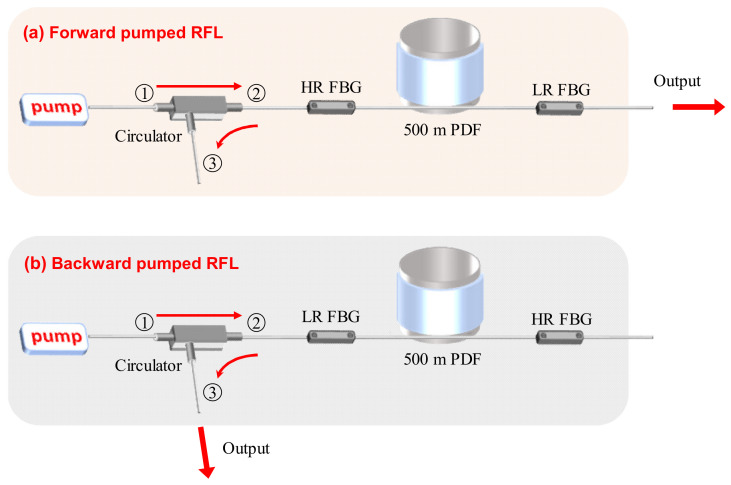
The experimental setups of (**a**) forward-pumped low-quantum defect Raman fiber laser and (**b**) backward-pumped low-quantum defect Raman fiber laser. HR, high-reflective; LR, low-reflective; FBG, fiber Bragg grating.

To verify the backward-pumped low-QD RFL’s advantage in suppressing the Raman-assisted FWM effect as well as spontaneous Raman generation at 14.7 THz, we built a forward-pumped RFL and a backward-pumped RFL. The experimental setups of these two low-QD RFLs are shown in Figure 3. They adopt the same fiber components. The pump source is a tunable laser source which can deliver 50 W output power over a tuning range of 1055–1075 nm. The Raman gain medium is a section of 500 m phosphosilicate fiber, the transmission loss of which is 1.56 dB/km at 1080 nm. A pair of 1080 nm fiber Bragg gratings (FBG) are adopted to form an oscillation cavity. The reflectivity of the high-reflective FBG and low-reflective FBG is about 99.5% and 10% respectively, and their bandwidths are 1.59 nm (high reflective FBG) and 1.05 nm (low reflective FBG), respectively. The pump source is coupled into the oscillation cavity through a circulator, which could separate the backward light from the pump source. The circulator has a broad bandwidth of 1060–1100 nm. The insertion losses from port one to port two and port two to port three are 0.36 dB and 0.31 dB, respectively. To be noticed, the pigtail fibers of the pump source, circulator and the two FBGs have the same core diameter of 10 μm, but the core diameter of this phosphosilicate fiber is 5 μm. Due to the mismatch of mode field diameter, there is a splicing loss of 0.43 dB between the pigtail fiber of the FBG and phosphosilicate fiber. In the forward-pumped RFL, port two of the circulator is spliced with the high-reflective FBG and the free end of the low-reflective FBG is the output port. In the backward-pumped RFL, port two of the circulator is spliced with the low-reflective FBG and port three of the circulator is the output port. Both output ports of these two lasers are cleaved at an angle of 8° to suppress the Fresnel reflection.

## 4. Results and Discussion

**Figure 4:**
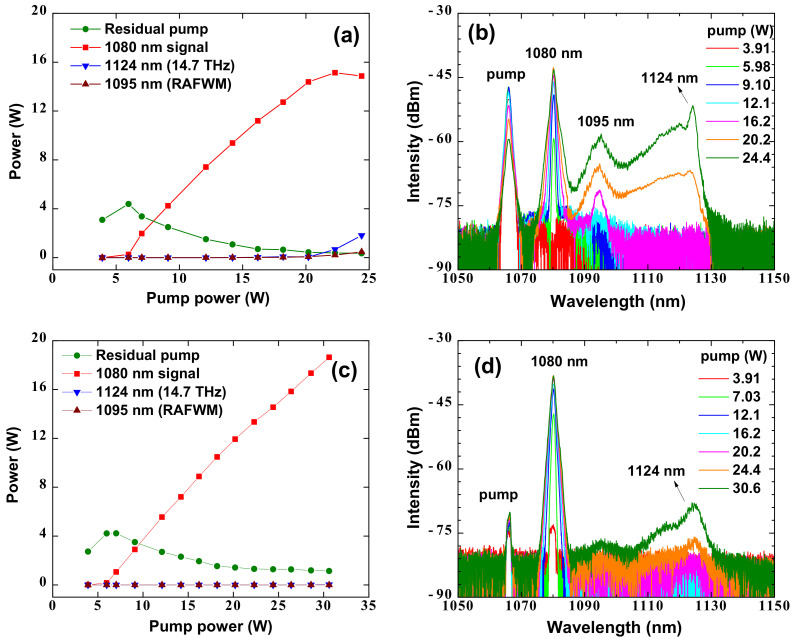
(**a**)The power evolution of the forward-pumped low-quantum defect Raman fiber laser, (**b**) the spectra evolution of the forward-pumped low-quantum defect Raman fiber laser, (**c**) the power evolution of the backward-pumped low-quantum defect Raman fiber laser, (**d**) the spectra evolution of the backward-pumped low-quantum defect Raman fiber laser. RAFWM, Raman-assisted four-wave mixing.

Considering that the frequency shift of the boson peak in the phosphosilicate fiber we adopt is 3.65 THz [22], the pump wavelengths in these two low-QD RFLs are set at 1066 nm for maximum Raman gain. The output power and spectra evolution of the forward-pumped low-QD RFL are shown in Figure 4a,b, respectively. Obvious threshold characteristics of the stimulated Raman scattering effect are observed. Once the pump power reaches the threshold of 5.98 W, the pump power is converted into the 1080 nm signal light rapidly. When the pump power increases to 16.2 W, a new peak at around 1095 nm is observed, which is the result of the Raman-assisted FWM effect. Subsequently, another peak at 1124 nm is generated under a pump power of 20.2 W, which is contributed by the spontaneous Raman generation at 14.7 THz. After the pump power increases to 22.3 W, the signal power reaches a maximum of 15.1 W, corresponding to a conversion efficiency of 67.7%. The spectral purity of signal is 94.2%. When the pump power is further increased to 24.4 W, the output power of the 1080 nm signal light is decreased to 14.8 W, while the power of the 1095 nm and 1124 nm lights is increased to 0.47 W and 1.81 W, respectively. The further power increase of the 1080 nm signal light is limited by the Raman-assisted FWM effect and spontaneous Raman generation at 14.7 THz. Figure 4c,d show the output power and spectra evolutions of the backward-pumped low-QD RFL. Similar to the forward-pumped low-QD RFL, the 1080 nm output signal is generated under a threshold power of 5.98 W. However, with the further increase in the pump power, no apparent Raman-assisted FWM effect is observed in the backward-pumped low-QD RFL. Only after the pump power increases to 30.6 W, a small spontaneous Raman peak at 1124 nm is generated. The corresponding output power of the 1080 nm signal light is 18.6 W, and its spectral purity is up to 99.8%. The further power increasing of the 1080 nm signal light is restricted by the power handling capacity of the circulator. We believe that higher output power can be achieved providing with a higher power circulator or wavelength division multiplexer which could separate the backward signal light from the pump. To be noticed, in the backward-pumped low-QD RFL, the power conversion efficiency under maximum output power is 60.8%, lower than the 67.7% of the forward-pumped RFL. This could be the result of incomplete Raman conversion and extra insertion loss from port 2 to port 3 in the circulator.

**Figure 5:**
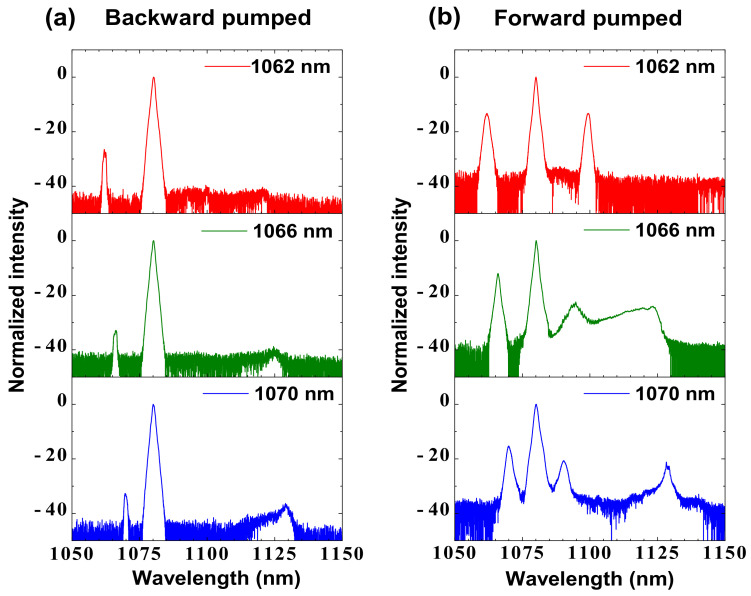
(**a**) The output spectra of the backward-pumped low-quantum defect Raman fiber laser under different pump wavelengths and the same pump power. (**b**) The output spectra of the forward-pumped low-quantum defect Raman fiber laser under different pump wavelengths and the same pump power of 20.2 W.

To further confirm the backward-pumped low-QD RFL’s advantage in suppressing the Raman-assisted FWM effect and spontaneous Raman generation at 14.7 THz, we compare the output characteristics of these two RFLs under three different pump wavelengths (1062, 1066, 1070 nm). Figure 5 shows the corresponding output spectra under the same pump power of 20.2 W. It can be seen that obvious Raman-assisted FWM effect related peaks are observed in the forward-pumped low-QD RFL under different pump wavelengths. However, there are no any signs of the Raman-assisted FWM process in the backward-pumped low-QD RFL under these pump wavelengths. Moreover, in the forward-pumped RFL, the spontaneous Raman generation at 14.7 THz is much stronger than that of the backward-pumped RFL. These results could further prove the backward pump scheme’s advantage in suppressing the Raman-assisted FWM effect and spontaneous Raman generation at 14.7 THz in the phosphosilicate fiber-based low-QD RFL.

## 5. Conclusions

In conclusion, the nonlinear process in the low-QD RFL is comprehensively studied theoretically and experimentally. A revised power-balanced model is proposed to simulate the gain competition process between the signal light, Raman-assisted FWM-generated light and spontaneous Raman generation at 14.7 THz in the low-QD RFL. The power evolution characteristics under different pump directions are calculated. The simulation results show that compared to the forward-pumped low-QD RFL, the threshold powers of spontaneous Raman generation at 14.7 THz and Raman-assisted FWM generated light in the backward pumped RFL are increased by 40% and 50%, respectively. Based on the simulation work, we change the pump direction of a forward-pumped low-QD RFL into backward pumping. As a result, the maximum signal power is increased by 20% and the corresponding spectral purity is increased to 99.8%. This work offers a way for nonlinear effect controlling in low-QD RFL, which is essential in its further performance scaling.

## Figures and Tables

**Table 1:** Fiber parameters in our numerical calculation.

Symbol	Value
*λ*_0_, *λ*_1_, *λ*_2_, *λ*_3_,	1066, 1080, 1124, 1095 nm
*α*_0_, *α*_1_, *α*_2_, *α*_3_,	0.44, 0.41, 0.34, 0.38 km^−1^
*g_R_*_01_, *g_R_*_02_, *g_R_*_03_, *g_R_*_12_, *g_R_*_13_	0.6, 1, 0.5, 0.8, 0.6 (×10^−13^ m/W)
*L*	500 m
*A_eff_*	33.2 μm^2^
*n*_2_	2.3 × 10^−20^

## Data Availability

The data are available on reasonable request from the corresponding author.
